# Topical Nanoemulgel for the Treatment of Skin Cancer: Proof-of-Technology

**DOI:** 10.3390/pharmaceutics13060902

**Published:** 2021-06-18

**Authors:** Sreeharsha Nagaraja, Girish Meravanige Basavarajappa, Mahesh Attimarad, Swati Pund

**Affiliations:** 1 Department of Pharmaceutical Sciences, College of Clinical Pharmacy, King Faisal University, Al-Hofuf, Al-Ahsa 31982, Saudi Arabia; mattimarad@kfu.edu.sa; 2 Department of Pharmaceutics, Vidya Siri College of Pharmacy, Off Sarjapura Road, Bangalore 560035, Karnataka, India; 3 Department of Biomedical Sciences, College of Medicine, King Faisal University, Al-Hofuf, Al-Ahsa 31982, Saudi Arabia; gmeravanige@kfu.edu.sa; 4 Nanomedicine Laboratory, Department of Biosciences and Bioengineering, Indian Institute of Technology-Bombay, Mumbai 400076, Maharashtra, India; swatipund@iitb.ac.in

**Keywords:** self-emulsifying, chrysin, ex vivo permeation, nanoemulgel, in vitro cytotoxicity, ex vivo permeation

## Abstract

The present study is a mechanistic validation of ‘proof-of-technology’ for the effective topical delivery of chrysin nanoemulgel for localized, efficient treatment of melanoma-affected skin. Background: Currently available treatments for skin cancer are inefficient due to systemic side effects and poor transcutaneous permeation, thereby presenting a formidable challenge for the development of novel nanocarriers. Methods: We opted for a novel approach and formulated a nanocomplex system composed of hydrophobic chrysin dissolved in a lipid mix, which was further nanoemulsified in Pluronic^®^ F-127 gel to enhance physicochemical and biopharmaceutic characteristics. Chrysin, a flavone extracted from passion flowers, exhibits potential anti-cancer activities; however, it has limited applicability due to its poor solubility. Pseudo-ternary phase diagrams were constructed to identify the best self-nanoemulsifying region by varying the compositions of oil, Caproyl^®^ 90 surfactant, Tween^®^ 80, and co-solvent Transcutol^®^ HP. Chrysin-loaded nanoemulsifying compositions were characterized for various physicochemical properties. Results: This thermodynamically stable, self-emulsifying drug delivery system showed a mean droplet size of 156.9 nm, polydispersity index of 0.26, and viscosity of 9100 cps after dispersion in gel. Mechanical characterization using Texture Analyzer exhibited that the gel had a hardness of 487 g and adhesiveness of 500 g. Ex vivo permeation through rat abdominal skin revealed significant improvement in percutaneous absorption measured as flux, the apparent permeability coefficient, the steady-state diffusion coefficient, and drug deposition. In vitro cytotoxicity on A375 and SK-MEL-2 cell lines showed a significantly improved therapeutic effect, thus ensuring reduction in dose. The safety of the product was established through biocompatibility testing on the L929 cell line. Conclusion: Aqueous, gel-based, topical, nanoemulsified chrysin is a promising technology approach for effective localized transcutaneous delivery that will help reduce the frequency and overall dose usage and ultimately improve the therapeutic index.

## 1. Introduction

The oral route is the most preferred route of drug delivery for the treatment of chronic diseases [1]. However, oral delivery of almost 50% of drugs is hampered because of high lipophilicity [2]. Nearly 40% of new drug candidates exhibit low solubility in water, which often leads to poor oral bioavailability and lack of dose proportionality [3]. The topical route for the management of skin disorders offers several advantages over the oral route and has tremendous potential for successful drug delivery. Nevertheless, the penetration of therapeutics through the skin suffers from a fewer side effects compared to oral drug administration [4]. Thus, there is a need for non-toxic and controlled drug delivery tools for effective topical delivery. Chrysin, a flavone [5], is one of the potential polyphenols widely distributed in plant-based foods [6]. A plethora of evidence supports the health benefits of polyphenolics in managing physiological functions and the prevention of chronic oxidative stress-triggered disease conditions such as cancers, diabetes, and cardiovascular as well as neurodegenerative disorders [7]. Chrysin, a bioactive phytoconstituent, has a broad range of pharmacological spectra showing anti-inflammatory, anti-oxidant, anti-aging, anti-cancer, and cardioprotective properties [8]. In addition, chrysin was found to be protective, non-toxic, and compatible with the human body [9]. However, chrysin is poorly water-soluble and has poor bioavailability [10]. Thus, there is a need to explore the routes through which the solubility and bioavailability for a specific disease segment could be enhanced. Nanotechnology offers new opportunities for improving the physicochemical properties of pharmaceutical molecules. Particle size reduction [11], one of the approaches to enhance the dissolution rate of a drug, may not be desirable for poorly wettable substances. Various other explored strategies are cyclodextrin complexation, solid dispersions, and self-emulsifying drug delivery systems [12,13,14]. Self-emulsifying drug delivery systems are isotropic mixtures of oils, surfactants, solvents, and co-solvents/surfactants that improve the physicochemical and pharmacological properties of highly lipophilic drug compounds [15,16,17]. Despite the potential of self-emulsifying formulations, their applicability for topical delivery is still limited, possibly due to less discovered skin metabolomics and physical stability. Modulation of the lipid arrangement of the stratum corneum is easily achieved by the oils and surfactants present in topical, self-emulsifying drug delivery systems. Furthermore, they also facilitate solubilization and partitioning of lipophilic actives in the skin layers [18,19]. To improve adhesion, the viscosity of nanoemulsion is increased by the addition of a gelling agent to form a nanoemulgel, which makes it easy and convenient for transcutaneous application.

The present study is a mechanistic approach to validate ‘proof-of-technology’ for the effective topical delivery of the bioactive phytoconstituent chrysin for the efficient, localized treatment of melanoma-affected skin regions. We supported our hypothesis with the physicochemical characterization of a nanoemulsifying drug delivery system, followed by mechanical characterization using a rheological force–time relationship. Percutaneous skin deposition; in vitro cytotoxicity in skin cancer cells, namely A375 and SK-MEL-2; and biocompatibility strengthened our ‘proof-of-technology’.

## 2. Materials and Methods

Chrysin was purchased from Sigma-Aldrich, Inc., India. Acrysol^®^ K-140 and Acrysol^®^ K-150 (propylene glycol–polyoxyl 40–hydrogenated castor oil) were gifted by Coral Pharma Chem (Ahmedabad, India). Capmul^®^ MCM (glyceryl caprylate/caprate) and Captex^®^ 355 were gift samples from Abitech Corporation, OH, USA. Capryol^®^ 90 (propylene glycol monocaprylate), Labrafac^®^ CC (caprylic capric triglyceride), and Labrasol^®^ (caprylocaproyl macrogol-8 glycerides) were gifted by Gattefosse India Pvt., Ltd. (Mumbai, India). Tween^®^ (polysorbate) 20 and Tween^®^ (polysorbate) 80 were purchased from Molychem (Mumbai, India). Cremophor^®^ EL, Cremophor^®^ RH 40, and Pluronic^®^ F127 were received as gift samples from BASF (Mumbai, India). Polyethylene glycol 400 and propylene glycol were purchased from Loba Chemie, Ltd. (Mumbai, India). Ethanol (96% *v*/*v*) was purchased from Research Lab Fine Chem Industries (Mumbai, India). All these chemicals were of analytical grade and used without any further purification. 3-(4,5-dimethylthiazol-2-yl)-2,5-diphenyl tetrazolium bromide (MTT), Dulbecco’s modified Eagle’s medium (DMEM), Eagle’s minimum essential medium (EMEM), fetal bovine serum, antibiotic solution (100X), trypsin-EDTA solution, and Hank’s balanced salt solution (HBSS) were purchased from Himedia, India.

### 2.1. Methods

#### 2.1.1. Formulation of Chrysin-Loaded, Self-Nanoemulsifying Preconcentrate

The solubility of chrysin was determined in various oils/modified oils (Acrysol^®^ K150, Acrysol^®^ K140, Captex^®^ 335, Capmul^®^ MCM, Capryol^®^ 90, and Labrafac^®^ CC), surfactants (Cremophor^®^ EL, Cremophor^®^ RH 40, Labrasol^®^, Tween^®^ 20, and Tween 80) and co-solvents (ethanol, polyethylene glycol 400, propylene glycol, and Trancutol^®^ HP) using the shake flask method [20]. An excess of chrysin was added to 1 mL of the above-mentioned vehicles, and the resulting suspensions were shaken on a flask shaker (Kytose EOS-10M, Electrolab) at room temperature for two days and centrifuged at 3000 rpm for 15 min (Spinwin MC 01, Tarson, Mumbai, India). The clear supernatant was analyzed for its content of chrysin using the validated RP-HPLC method later described in the analytical methods section. Determinations were carried out in triplicate.

Based on the solubility data, a series of self-nanoemulsifying preconcentrates were prepared comprising Capryol^®^ 90 as the oil phase, Tween^®^ 80 as the surfactant, and Transcutol^®^ HP as the co-solvent. The concentrations of oil (surfactant and co-solvent; Smix) were varied from 1:9 to 9:1 whereas surfactant proportions varied at 1:1, 1:2, and 2:1. Chrysin 100 mg/mL was added to all preconcentrates and was thoroughly mixed for 30 min. The influence of aqueous dilution on these anhydrous, chrysin-loaded preconcentrates was analyzed. The mixtures were titrated slowly with aliquots of distilled water and visually monitored for ease of emulsification and homogeneity.

#### 2.1.2. Conversion of Self-Nanoemulsifying Concentrate into a Nanoemulgel for Topical Application

The gel was formulated by dispersing the self-nanoemulsifying preconcentrate in water containing the gelling agent [18,19,21]. Pluronic^®^ F127 (20% *w*/*w*) was dissolved in cold water. Chrysin-nanoemulsifying preconcentrate (10% *v*/*w*) containing chrysin 100 mg/mL was added to the clear solution of Pluronic^®^ F127 at 10 °C to get chrysin concentration 1% *w*/*w*. The resulting mixture was sonicated in an ultrasonic water bath for 5 min to remove the entrapped air. For comparative study, another gel consisting of a dispersion of chrysin (1% *w*/*w*) was also prepared by thoroughly dispersing the equivalent amount of chrysin in Pluronic^®^ F127 gel.

#### 2.1.3. Characterization of Chrysin-Loaded Self-Nanoemulsifying Drug Delivery System

Droplet size analysis, polydispersity index, Zeta potential, and electron microscopy of the nanoemulsion: The chrysin-loaded, self-nanoemulsifying drug delivery system was diluted with water (1:10) to form a nanoemulsion [12,13,14]. The resultant nanoemulsion was subjected to characterization of the droplet size and polydispersity index using photon cross-correlation spectroscopy. Formulations were diluted with double-distilled water to ensure that the light scattering intensity was within the instrument’s sensitivity range. Each sample was placed in a transparent polystyrene cuvette (path length = 1 cm) and placed in a thermostatic sample chamber. Mean droplet size and the span of the resulting emulsions were determined by photon cross-correlation spectroscopy (Nanophox, Sympatec, Clausthal-Zellerfeld, Germany). Detection was carried out at a scattering angle of 90°. From the resulting correlation curves, a 2nd order analysis was performed to calculate the mean droplet size. The droplet size distribution was expressed in terms of the polydispersity index. Zeta potential was measured on a zeta meter (Delsa Nano C, Beckman Coulter, Tokyo, Japan). Measurements were carried out immediately after reconstitution as well as 24 h and 48 h after storing the sample at ambient temperature.

For scanning electron microscopy (SEM), the diluted nanoemulsion (10 μL) was placed on carbon-conductive adhesive tape mounted on the specimen stub. The mounted sample was frozen at −190 °C in liquid nitrogen and transferred to the preparation chamber, maintained at −130 °C, and sublimed at −90 °C for 10 min, followed by coating with platinum. It was then transferred to the SEM chamber for viewing at −150 °C with an accelerating voltage of 5.0 kV (JSM-7600F Field Emission Gun (FEG) SEM equipped with Cryo unit (PP3000T) by Quorum). For transmission electron microscopy (TEM), the nanoemulsion was mounted on a carbon-coated formvar grid and stained with neutralized 2% phosphotungstic acid. The grid was dried and imaged on high-resolution TEM (JEM 200, JEOL).

Thermodynamic stability: The nanoemulsifying preconcentrates were tested for 3 cycles of heating and cooling at 45 °C and 4 °C, respectively, with a storage time of 48 h at each temperature. The product was observed visually for precipitation and centrifuged for 10 min at 3000 rpm at every stage. Freeze–thaw stability was analyzed by storing the premix at 25 °C and −25 °C, with storage for 48 h at each temperature. The cloud point of the nanoemulsion obtained after the dilution of chrysin-loaded preconcentrate with water was also determined by heating the emulsion gradually until turbidity or phase separation was observed.

#### 2.1.4. Analysis of Chrysin by RP-HPLC

The RP-HPLC method for estimating chrysin content was validated for accuracy, precision, specificity, and solution stability. The method was found to be specific, as observed from the absence of any interfering peaks at the retention time of the analyte. The peak purity of chrysin was 99.99%. The method was found to be linear, in a concentration range of 0.025 to 10 μg/mL, precise (%CV: 1.05–1.36), and accurate (98.9–101.8%). The limit of detection and the limit of quantification were 0.01 and 0.025 μg/mL, respectively. The solution-state stability of chrysin in its mobile phase and phosphate-buffered saline was within an acceptable range (98.5–101.5%) at 20 °C for 1 month and 4 °C for 3 months, respectively.

All test samples were diluted suitably with mobile phase, and chromatographic separation was performed using an isocratic elution. The mobile phase consisted of a mixture of buffer and acetonitrile (45:55) and was delivered at a flow rate of 1 mL/min. The HPLC system consisted of a pump (Jasco PU-2080 Plus, Intelligent HPLC pump, Tokyo, Japan) connected to a detector (Jasco 2075, Intelligent UV–vis detector, Tokyo, Japan). The separation was carried out at 20 °C on a reversed phase C8 column (Agilent, 150 × 4.6 mm, 5 μm particle size). An injection volume of 20 μL was used. Detections were carried out at 270 nm.

#### 2.1.5. Characterization of the Nanoemulgel of Chrysin for Topical Delivery

##### Droplet Size, Polydispersity Index, Electron Microscopy, and Viscosity

The gel sample was diluted with water (1:100) and analyzed for droplet size as per the method described above for the nanoemulsifying drug delivery system. The nanoemulgel was also imaged for SEM in cryo-mode, as mentioned earlier. The droplet size of the nanoemulgel was measured over a period of 3 months to analyze the effects on size and size distribution.

The viscosity of the gel was measured at varying shearing rates at room temperature on a Brookfield viscometer (DV-II+ Pro, Brookfield Engineering Labs., Inc., USA) equipped with a helipath stand and T-bar spindle.

##### Mechanical Properties of the Gel Using Texture Profile Analyzer

A texture analyzer (CT3, Brookfield Engineering, Middleboro, MA, USA) equipped with 10 kg load cell was used for complete mechanical testing of the nanoemulgel. The nanoemulgel was placed into the cone of the spread test fixture and allowed to set at room temperature for 15 min. The excess formulation was scraped off from the cone holder to get a smooth and flat surface for the analysis and avoid early triggers for the test. The probe (TA3/100, 45°, 30 mm diameter) was programmed in texture profile analysis mode to move into the gel with a pre-test speed of 1 mm/s and test speed of 0.5 mm/s, and it was moved out of the gel at a return speed of 0.5 mm/s. The test was performed in 2 cycles, with an acquisition rate of 100 points/s. The data was collected and analyzed using TexturePro CTv1.2 (Middleboro, MA, USA) software to determine various mechanical performance properties such as hardness, cohesiveness, and springiness.

##### Ex Vivo Percutaneous Permeation and Skin Retention

The experimental protocol was approved by the Institutional Animals Ethics Committee, and the experiment was carried out according to the guidelines of the Committee for the Purpose of Control and Supervision of Experiments on Animals (CPCSEA) for experimental animal care. Rats weighing 200–220 g were housed in standard wire mesh plastic cages in a room maintained at 22 ± 0.5 °C and a day/night cycle of 12 h each. Animals were given standard pellet food and water ad libitum. After anaesthetizing the male Wistar rats with ether, the hairless abdominal skin was surgically removed. The adhering subcutaneous fat was carefully removed, and skin was mounted on vertical, static, jacketed Franz-type diffusion cell with a diffusion area of 3.14 cm^2^, with phosphate-buffered saline pH 7.4 (32 °C) in the receptor chamber. Skin was allowed to stabilize for 15 min. Chrysin nanoemulgel (1 g) was placed in the donor side of the diffusion cell. Samples from the receptor chamber (200 μL) were withdrawn at periodic time intervals and analyzed for amount of chrysin permeated, using the validated RP-HPLC method. A similar experiment was also performed for the simple gel of chrysin dispersed in gel base. Various kinetic parameters were derived from the data obtained and the steady-state flux (Jss, mg/cm^2^ × h). The apparent permeability coefficient (Papp, /cm^2^ × min) and steady-state diffusion coefficient (D) were calculated. After completion of the permeation studies, the treated skin mounted on the diffusion cell was removed carefully and homogenized with acetonitrile to determine the chrysin deposited in the skin [22].

#### 2.1.6. In Vitro Cytotoxicity of Chrysin Nanoemulgel on A375 and SK-MEL-2 Cell Lines

The in vitro cytotoxicity study of the chrysin nanoemulgel was analyzed on human melanoma A375 and SK-MEL-2 cell lines. A375 and SK-MEL-2 cells were cultured in Dulbecco’s modified Eagle medium (DMEM), and Eagle’s minimum essential medium (EMEM), respectively. The medium was supplemented with 10% fetal bovine serum and 100 U/mL penicillin and streptomycin. Cells were maintained at 37 °C in a humidified atmosphere with 5% carbon dioxide. Cell lines were trypsinized and added to the 96-well tissue culture plates. The cell suspension was added to 96-well tissue culture plates (1 × 10^4^ cells per well) and incubated overnight for cell attachment. Following the overnight cell attachment, the medium was replaced with complete medium (100 µL) containing pristine chrysin and the developed chrysin nanoemulgel. The cells were incubated with chrysin concentrations in the range of 10 to 40 µg/mL for 48 h, and cell viability was determined using MTT assay. These experiments were performed in triplicate, and the cell viability was given as mean ± standard error of mean (*n* = 3). The growth inhibition factor was calculated.

#### 2.1.7. In Vitro Biocompatibility Analysis

The nanoemulgel of chrysin was tested for cell viability in the mouse fibroblast cell line (L929). The cells were maintained in DMEM and distributed in 96-well plates (1 × 10^4^ cells/well) for 24 h in a 5% CO_2_ atmosphere at 37 °C. After this period, the medium was removed, and the adhered cells were treated with formulations (4 dilutions, using 0.2 g/mL of formulation in DMEM as 100% and further dilution with DMEM to obtain 50%, 25%, and 12.5%, *n* = 6) under the same incubation conditions. Untreated cells were used as controls and their growth was considered 100% cell viability. The viability was determined by MTT assay as described in ISO 10993-5. MTT (1 mg/mL in HBSS) was added to each well of the plate, which was again incubated for 3 h in a 5% CO_2_ atmosphere at 37 °C. After this, the medium was aspirated, and the formazan crystals formed were dissolved in dimethyl sulfoxide. After 30 min, the optical density was measured on a microplate reader at a wavelength of 570 nm, and the percentage of viability was expressed as the growth rate (%) in comparison to the control.

## 3. Results

We developed thermodynamically stable, lipid-based, nanoemulsifying preconcentrates of chrysin, ready for reconstitution in a gel base. A very simple, easily scalable, self-nanoemulsifying drug delivery system offers a promising approach to facilitating the delivery of lipophilic drugs through nanosizing into an aqueous gel that aids their deep delivery through the skin matrix.

### 3.1. Formulation Design

For the successful delivery of drugs via a self-nanoemulsifying drug delivery system, the entire dose of the drug must be soluble in an acceptable volume of lipid mix. If the drug solubility is inadequate, there is a likelihood of drug precipitation upon aqueous dilution. In addition, the probability of drug precipitation increases when co-solvents contribute greatly to the overall drug solubility. Co-solvents such as PEG, propylene glycol, and alcohols help to solubilize large quantities of the drug in oil. Upon aqueous dilution, the co-solvents separate from the oil components, forming a micellar dispersion that results in the reduction of solvent capacity for the drug [22]. Thus, the solubility of the drug in the excipients is an important criterion for successful formulation development. Accordingly, the equilibrium solubility of chrysin was estimated in lipids, surfactant, and co-solvents.

**Figure 1:**
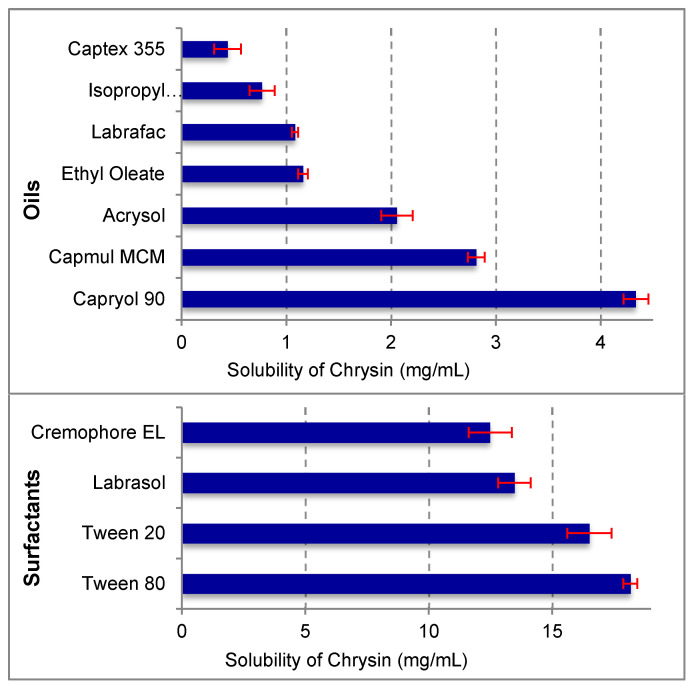

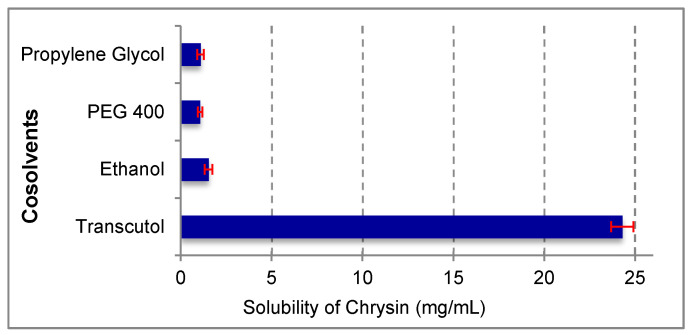
Solubility of chrysin in various oils, surfactants, and co-solvents (*n* = 3 ± SD).

Chrysin showed the highest solubility in Capryol^®^ 90 (4.34 mg/mL), Tween^®^ 80 (18.16 mg/mL), and Transcutol^®^ HP (24.3 mg/mL) among the oils, surfactants, and cosolvents used, respectively (Figure 1). Therefore, these three formulation components were selected for further experimentation.

### 3.2. Preparation of Nanoemulsifying Preconcentrates

Among the three different combinations of surfactant and co-solvent evaluated, the Smix = 2:1 system had a higher capacity to solubilize much of the oily phase. The branched structure of the surfactant imparted better emulsification capacity, as it promoted greater penetration of the oil. The uniform droplet size distribution and emulsion region alluded to the higher emulsifying potential of the preconcentrate combination. It is always desirable to have a formulation with better emulsifying ability even with a lower proportion of surfactant and also have higher drug loading potential [23]. In addition, the smaller the difference between the chain length of the surfactant and the oil, the better the self-nanoemulsification. Hence, Smix at a 2:1 ratio was optimized as the emulsifying preconcentrate for chrysin loading.

### 3.3. Physicochemical Characterization of the Nanoemulsifying Drug Delivery System

#### 3.3.1. Droplet Size, Polydispersity Index, Zeta Potential, and Electron Microscopy

**Figure 2:**
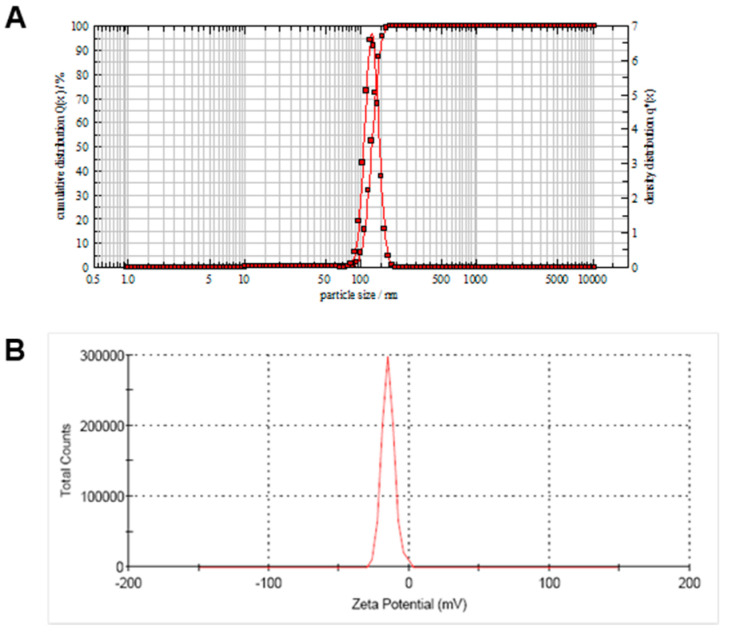
Characterization of nanoemulsion of chrysin obtained after dilution of nanoemulsifying preconcentrate with water. (**A**) Particle size measured using photon cross-correlation spectroscopy and (**B**) Zeta potential.

The droplet size was found to be less than 300 nm for the entire series of formulations prepared with Capryol^®^ 90, ranging from 10 to 90%. Droplet size was found to increase with the increase in the oil content of the formulation. The mean droplet size of the formulation with Capryol^®^ 90: Smix, 8:2 was found to be 123.4 ± 5.4 nm (*n* = 3 ± SD; Figure 2A), and the polydispersity index was 0.26, indicative of a narrow droplet size distribution. Droplet size and PDI showed no significant changes over 3 months when stored at ambient temperature. No significant change in size was observed when the samples were stored at ambient temperature for 24 h and 48 h.

Small droplet size implied faster release from the formulation due to increased surface area and the higher probability of an enhanced rate of chrysin penetration through the skin and absorption into the cells. The surface charge of the nanoemulsion formed from the aqueous dispersion of the self-nanoemulsifying drug delivery system played a vital role in its stability [24]. Using Capryol^®^ 90 as the oil and Tween^®^ 80 as the surfactant with co-solvent Transcutol^®^ HP, the emulsion obtained was negatively charged with a zeta potential of −15 mV (Figure 2B). The negative charge on the emulsion droplets indicated the presence of anionic fatty acids and glycols in the oil and surfactant [25]. For small molecules, the stability was considered high if the zeta potential value was high and negative whereby the droplets opposed the aggregation. However, the current technology involved additional steric stabilization of the emulsion droplets in a semisolid gel base.

**Figure 3:**
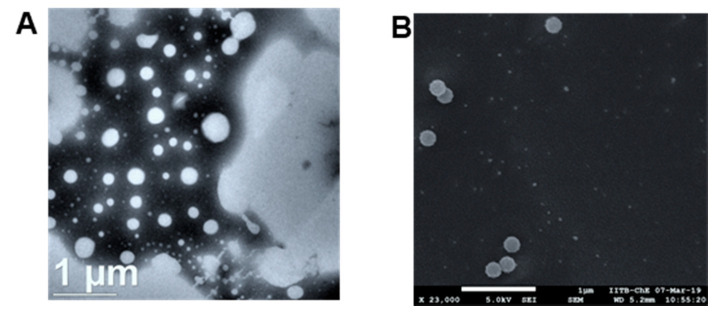
Electron microscopy images of chrysin-loaded, nanoemulsifying preconcentrate after dilution with water. (**A**) TEM image and (**B**) SEM image.

SEM and HR-TEM images of the nanoemulsion formed after dilution of the nanoemulsifying preconcentrate of chrysin are shown in Figure 3A,B. Both images show uniform size distribution and spherical shape.

#### 3.3.2. Thermodynamic Stability of the Chrysin Nanoemulsifying Concentrate and the Nanoemulsion Obtained after Dilution

The cloud point is an essential factor in self-nanoemulsifying drug delivery systems consisting of non-ionic surfactants, as it is an indicator of the successful formation of a stable emulsion. When the temperature is higher than the cloud point, an irreversible phase separation occurs. The cloudiness of the preparation adversely affects drug absorption because of the dehydration of the components. Hence, it is desirable to have the cloud point for self-nanoemulsifying drug delivery systems above 37 °C to avoid phase separation in vivo in the gastrointestinal tract [23]. The cloud point for the developed formulation was greater than 65 °C, suggesting the formulation was stable at body temperature, i.e., 37 °C. In self-emulsifiable drug delivery systems, the interfacial tension is made sufficiently low such that interfacial energy becomes comparable or even lower than the entropy of dispersion, and hence, the free energy of the system becomes zero or negative [26]. This explains the thermodynamic stability of the emulsion. The thermodynamic stability of the prepared formulations was studied to avoid the occurrence of any metastable formulations. The metastable emulsions, upon contact with biological fluid, might lead to instability and non-effectiveness of the formulation. The chrysin-loaded emulsifying system showed no precipitation or phase separation, thus suggesting its stability.

### 3.4. Evaluation of Chrysin-Loaded Nanoemulgel for Topical Delivery

#### 3.4.1. Droplet Size and Viscosity

**Figure 4:**
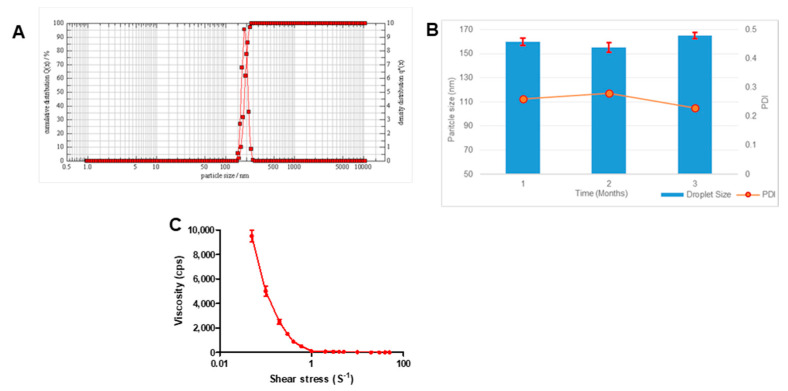
Characterization of chrysin-loaded nanoemulgel. (**A**) Representative graph showing droplet size, (**B**) bar graph showing droplet size and line graph showing PDI over a period of 3 months, and (**C**) viscosity versus shear stress profile.

Transparent gels are easy and convenient for topical application. Pluronic^®^ F127 gel was used for reconstitution, as it provided several benefits over traditional ointments in terms of ease of application, non-occlusive application, drug release profile, and easy washability. The droplet size of the chrysin-loaded nanoemulgel was 156.9 ± 3.4 nm (*n* = 3 ± SD; Figure 4A).

This increase in size as compared to the size of droplets in the diluted emulsion was due to the adsorption of the gelling polymer over the emulsion droplets, providing additional stearic stability. However, there was no change in the polydispersity index. Stability analysis of the gel for 3 months showed no significant difference in droplet size or PDI (Figure 4B).

Viscosity measurements (Figure 4C) showed that the nanoemulgel exhibited shear-thinning property, as observed from decreases in viscosity with increases in the applied shear force. The viscosity reduced from 9500 ± 490 to 102 ± 12 cps with increasing shear stress from 0.05 to 10 (s^−1^). This non-Newtonian behavior of flow was ideal, as it facilitated extrusion of the gel through the tubes and ease of application.

#### 3.4.2. Mechanical Properties of the Gel, Using Texture Profile Analyzer

**Figure 5:**
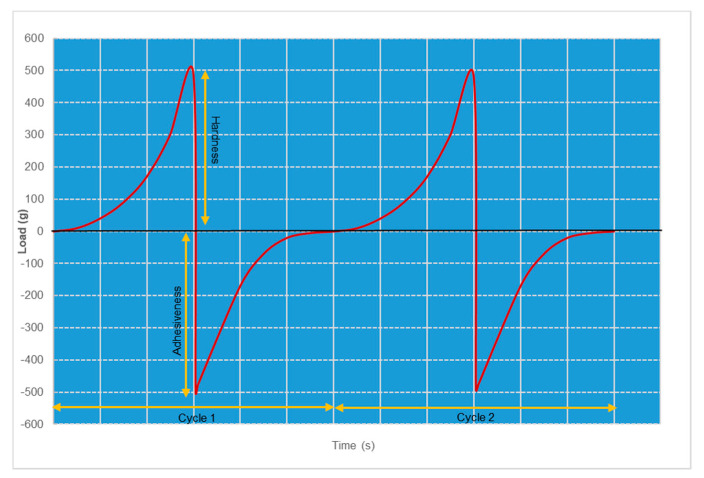
2 cycles of compression and decompression of the force–time texture profile of chrysin nanoemulgel, determined using texture analyzer.

For Pluronic^®^ hydrogels, the measurements of cohesiveness, adhesiveness, and hardness offered fast and reliable methods to characterize texture properties. Various factors such as the polymer concentration and the incorporation of drugs and additives significantly influenced texture properties [27]. Although hardness and adhesiveness were mechanical properties, they directly affected the therapeutic outcome of the product. The texture profile graph indicating the force–time relationship, with two cycles of compression and decompression for the chrysin-loaded nanoemulsifying gel, is shown in Figure 5.

Both cycles represent uniform and smooth curves, indicative of the smoothness of the gel. The hardness of the gel, which represented the strength of the gel structure under compression, was 489 and 485 g in the first and second cycles, respectively. Similarly, the adhesiveness of the gel was directly related to bioadhesion and thus ensured skin retention and prolonged activity.

#### 3.4.3. Ex Vivo Percutaneous Permeation and Skin Retention

Effective topical therapy needs a sufficient concentration of the drug crossing the stratum corneum and going deep into the skin. This is greatly influenced by the rate and the extent of the percutaneous penetration of the drug. The percutaneous penetration of chrysin through nanoemulgel was compared with that of simple gel prepared by dispersing chrysin powder in Pluronic^®^ gel base. Several derived parameters were calculated as the steady-state flux (Jss, mg/cm^2^ × h), the apparent permeability coefficient (Papp, /cm^2^ × min), and the steady-state diffusion coefficient (D). For comparison purposes, the initial concentration of chrysin in nanoemulgel, as well as in ordinary gel in the donor compartment, was kept as 10 mg, and the effective surface area available for permeation was 3.14 cm^2^. The data in Table 1 clearly indicate the improved percutaneous delivery of chrysin when delivered in nanoemulgel form.

When chrysin was dispersed in powder form in Pluronic^®^ gel, the penetration into the skin was slow and poor. Although chrysin had a higher log P, its extremely low aqueous solubility acted as the rate-limiting step for diffusion through the aqueous gel. In addition, the higher particle size of undissolved particles resulted in poor skin permeation. Solubilization of chrysin in lipid and surfactant enabled it to overcome the rate-limiting dissolution step, and delivery in nanosized droplets assisted in its rapid skin penetration.

### 3.5. In Vitro Cytotoxicity of Chrysin Nanoemulgel A375 and SK-MEL-2 Cell Lines

Chrysin is a natural and biologically active flavonoid with anti-cancer effects. Chrysin affects various molecular pathways and mechanisms including both intrinsic and extrinsic pathways of apoptosis in cancer therapy. Chrysin disrupts the homeostasis of mitochondria and endoplasmic reticulum E in apoptosis induction and also activates programmed cell death, or autophagy. The antiproliferative effect of chrysin has been reported in various cancer cell lines including melanoma cell lines, namely human A375 and A375.S2 and murine B16-F1 [28,29], A431 human epidermoid carcinoma cells [30], and the CT26 colon cancer cell line [28,31] have shown that chrysin inhibits MMP-2 activity and at concentrations lower than the lethal concentration, chrysin inhibits cell mobility, migration, and the invasion of cancer cells when analyzed using wound healing and Transwell filter assay. A recent review on the future perspectives of the broad-spectrum preclinical antitumor activity of chrysin has emphasized the need to formulate chrysin nanoparticles to overcome the potential drawback of its poor bioavailability [32].

**Figure 6:**
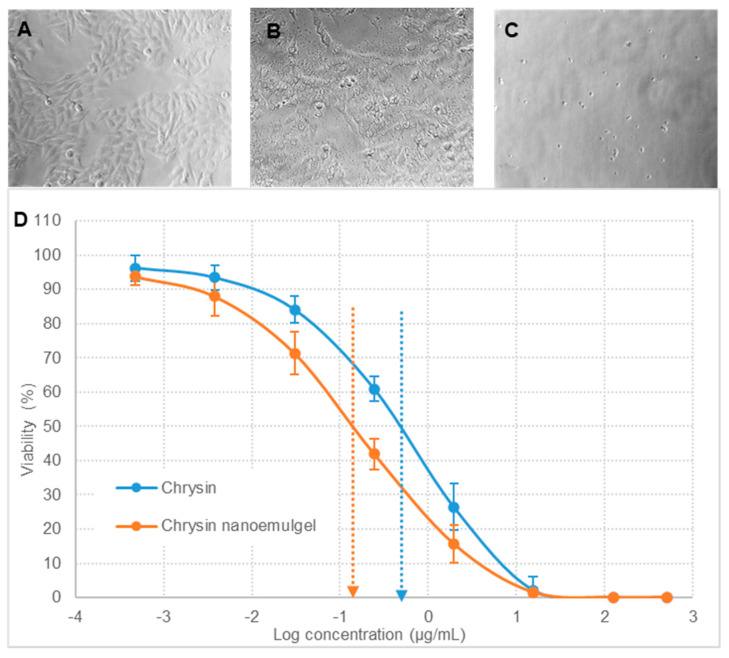
Morphological observation and growth inhibition of A375 cells. (**A**) control cells (untreated), (**B**) cells treated with pure chrysin, and (**C**) cells treated with chrysin nanoemulgel. Figure (**D**) represents the in vitro cytotoxicity profile, plotted as the viability of cells (%) versus the log of chrysin concentration (μg/mL). The data represented are mean values of 3 independent determinations, and error bars indicate standard error of triplicate analysis.

**Figure 7:**
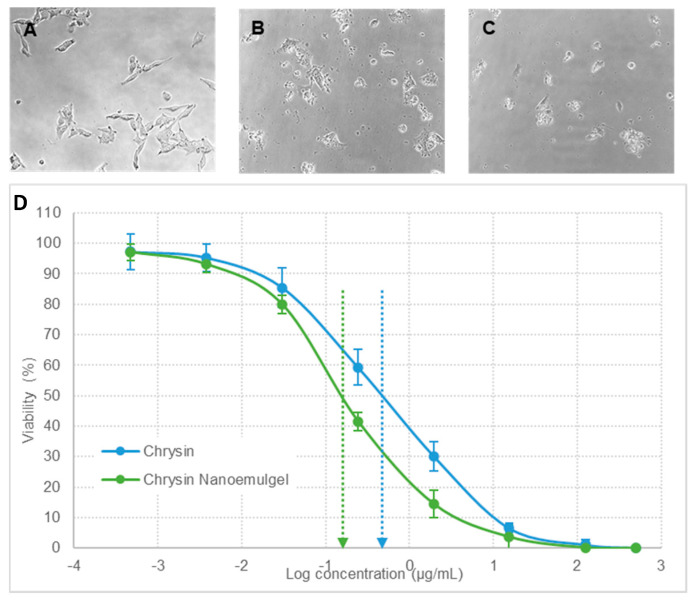
Morphological observation and growth inhibition of SK-MEL-2 cells. (**A**) control cells (untreated), (**B**) cells treated with pure chrysin, and (**C**) cells treated with chrysin nanoemulgel. Figure (**D**) represents the in vitro cytotoxicity profile, plotted as the viability of cells (%) versus the log of chrysin concentration (μg/mL). The data represented are mean values of 3 independent determinations, and error bars indicate standard error of triplicate analysis.

After being cultured and treated, the A375 and SK-MEL-2 cells were observed at periodic intervals, and changes in cell morphology were noted. Viability was analyzed using MTT assay [33]. Figure 4 and Figure 5 represent the in vitro cytotoxicity data of chrysin nanoemulgel in the A375 and SK-MEL-2 cell lines, respectively. Cells treated with pristine chrysin (Figure 6A and Figure 7A) retained normal cell morphology, whereas cells treated with chrysin powder (Figure 6B and Figure 7B) and nanoemulgel showed altered morphology and apoptotic changes such as rounding and shrinkage (Figure 6C and Figure 7C).

However, the effect was more profound in chrysin nanoemulgel treatment. Significant inhibition of the proliferation of A375 and SK-MEL-2 cells was observed when the cells were exposed to different concentrations of chrysin, ranging from 0.5 ng/mL to 500 µg/mL, and cells were monitored for up to 48 h. GI50, the concentration required to cause 50% growth inhibition, was calculated and was significantly lower for the chrysin nanoemulgel at 0.144 ± 0.04 µg/mL compared to chrysin-free drug treatment and 0.434 ± 0.11 µg/mL (*p* < 0.05) for the A375 cell line. Similarly, for the SK-MEL-2 cell line, GI50 was found to be 0.465 ± 0.14 and 0.169 ± 0.03 µg/mL for chrysin-free drug treatment and chrysin nanoemulgel, respectively (*p* < 0.05). These results indicate significant improvement in chrysin cytotoxicity against cancer cell lines after converting chrysin into a nanoemulgel.

### 3.6. In Vitro Biocompatibility Testing in the L929 Cell Line

The biocompatibility of the test formulation was analyzed by performing a cytotoxicity test and cell proliferation test on the L929 cell line [34]. For all four concentration levels of the nanoemulgel tested, the growth of cells was not affected. The viability of all four samples was >97% and hence, the formulation was considered biocompatible and safe for topical application.

## 4. Conclusions

The transcutaneous permeation of drugs through the keratinized stratum corneum is a major obstacle and challenge for topical delivery. In addition, currently available treatment for skin cancer suffers from numerous side effects. Thus, formulations that are skin permeable and compatible are of prime importance. Utilizing the medicinal properties of herbal components offers the benefit of developing formulations that are non-toxic, non-irritating, and compatible. Self-nanoemulsifying drug delivery systems loaded with chrysin have been successfully developed and explored for topical delivery in cancer treatments, especially for skin cancer. The physicochemical characterization demonstrated that the mean droplet size of the formulations was in nanoscale, with narrow size distribution and acceptable thermodynamic stability. Mechanical properties of the nanoemulgel measured as the force–time relationship using mechanical texture characteristics were optimal for its convenient and easy application to the skin surface. Ex vivo studies demonstrated that the nanoemulsified formulation significantly promoted the transcutaneous penetration and skin deposition of chrysin, justifying its utility for topical therapy. Cytotoxicity studies showed the enhanced therapeutic response of chrysin after conversion into nanoemulgel form. The results suggest that the prepared self-emulsifying drug delivery system is safe and biocompatible and will effectively reduce overall dose and chrysin consumption. Taking the improved physicochemical properties into account, the results of this study might open the door for numerous additional applications of the chrysin self-emulsifying drug delivery system for other applications including oral, nasal, and rectal delivery, imparting a new lease of life to the herbal nutraceutical. The formulation presents a versatile platform technology that can be optimized to incorporate a variety of hydrophobic, drug-loaded lipid nanocomplexes that will allow localized delivery of the therapeutic agent at the affected site. Versatility, prolonged skin retention, and avoiding systemic penetration are the unique advantages of the current platform technology applicable to skin diseases.

## Figures and Tables

**Table 1:** Various derived parameters for the percutaneous penetration of chrysin from nanoemulgel and simple gel.

Parameter	Chrysin-Loaded Nanoemulgel	Chrysin Dispersed in an Ordinary Gel
Steady state flux (Jss, mg/cm^2^ × h)	192.3 ± 10.9 *	27.8 ± 3.4
Apparent permeability coefficient (Papp, /cm^2^ × min)	29.6 ± 2.3 *	4.6 ± 1.23
Steady state diffusion coefficient (D)	1.89 ± 0.32 **	0.33 ± 0.04
Amount deposited in skin at 8 h	978.5 ± 63.5 **	167.6 ± 21.8

Each value represents the mean ± SD (*n* = 3). * *p* < 0.05, when compared with ordinary chrysin gel by Student’s *t*-test. ** *p* < 0.001, when compared with ordinary chrysin gel by Student’s *t*-test.

## Data Availability

All relevant data is included in the manuscript.
